# Social determinants and other aspects associated with rheumatic diseases in the Brazilian population: a cross-sectional study based on the National Health Survey (PNS2013)

**DOI:** 10.1186/s13690-020-00502-2

**Published:** 2020-11-16

**Authors:** Clécio Gabriel Souza, Marcelo Cardoso Souza, Hugo Jario Silva, Sanderson José Costa Assis, Diego Sousa Dantas

**Affiliations:** 1 Postgraduate program in rehabilitation sciences at the Federal University of Rio Grande do Norte, Santa Cruz, Brazil; 2 Public health at the Federal University of Rio Grande do Norte, Natal, Brazil; 3 Physiotherapy Federal University of Pernambuco, Recife, Brazil

**Keywords:** Chronic disease, Epidemiology, Epidemiological surveys, Cross sectional study, Rheumatic disease

## Abstract

**Background:**

Rheumatic diseases are increasingly present in the world population, represented by chronic joint and musculoskeletal pain. Among them, osteoarthritis (OA) is the most prevalent. It is considered the third most prevalent chronic non-communicable disease in the Brazilian population, being responsible for a high rate of physical disability and reduced quality of life. Little has been discussed about the social factors associated with this health condition. This study aimed to analyze the social factors associated with arthritis in the Brazilian population.

**Methods:**

This is a cross-sectional study based on data from the 2013 National Health Survey in Brazil with arthritis as its main outcome. Sex, age, body weight, usual activities, physical activity, self-perceived health and diagnosis of depression were analyzed as independent variables. Descriptive and inferential statistics were used. Poisson multiple regression was performed, and the prevalence ratio (PR) and confidence interval (CI) of 95% were calculated using a significance level of 5% (*p* ≤ 0.05).

**Results:**

A total of 60,202 individuals of both sexes took part in this study and the rheumatic diseases or arthrtitis prevalence was 6.4%. The individual factors associated with a higher prevalence of arthritis were female (PR = 2.09; CI = 1.95–2.25), age over 35 years (PR = 2.88; CI = 2.57–3.24) and excess body weight (PR = 1.61; CI = 1.25–2.07). The presence of rhemautic diseases showed an association with lower performance of usual activities (PR = 1.61; CI = 1.50–1.73) and self-perceived health as very poor (PR = 3.96; CI = 3.31–4.72). In addition, it was associated with a higher prevalence of mental illnesses such as depression (PR = 1.77; CI = 1.64–1.90).

**Conclusion:**

Social and modifiable factors which are associated with a higher prevalence of arthritis can be controlled through incentive measures such as social participation and physical activity.

## Bullet points

Little relevance is given about the social factors associated with rheumatic diseases and arthritis.

Rheumatic diseases such as arthritis are associated with poor performance in usual activities, poor self-perceived health and a greater presence of mental illnesses, such as depression.

Encouraging social participation and regular physical activity can improve the social and modifiable factors associated with arthritis.

## Background

Social determinants in health refer to a set of factors that influence all health problems and risk factors in a population. These factors involve individual, biological and social aspects, such as housing, education, socioeconomic level, as well as leisure, culture and public policies [1]. Studies such as the National Health Survey (PNS) are surveys that periodically reveal the health conditions and profile of the population.

Among the musculoskeletal diseases assessed by the PNS are rheumatic diseases or arthritis, with Ostearthritis (OA) being the most prevalent among them. This is a chronic-degenerative, progressive and multifactorial condition [2]. It commonly occurs in the aging process and affects several joints in the body, being more common in those responsible for weight support such as in the knees and hips [2, 3] as a mechanical and biochemical imbalance, which causes damage to the cartilage of joint surfaces and leads to inflammatory and repairing processes responsible for the appearance of bone deformities and physical disabilities [4]. It is the most common health condition in the population and the main cause of pain and disability in the world [2]. OA is among the 10 most disabling disease in developed countries, and its prevalence increases with advancing age, affecting about 75% of people over 65 years of age, and also being present in younger ages, especially in women with an average age of 50 years [5].

The consequences of arthritis go beyond joint pain and impairment, as it has a negative impact on the quality of life of individuals, especially in the social sphere [6]. It represents 65% of the causes of incapacity for work in Brazil, being considered the third cause of absences and sick leave [7] In addition, there is excessive expenditure on medications and varied treatments for this condition [8]. The sum of these factors results in important consequences for the country’s socioeconomic and health system [9, 10].

Most studies on the prevalence and factors associated with chronic non-communicable diseases (NCDs) in the Brazilian population [11–13] touch on OA by only estimating its prevalence, but without analyzing other factors associated with this chronic condition. Understanding these factors can contribute to elaborating specific public policies and promotion and prevention strategies which aim to reduce the prevalence and disabilities that can arise from the diagnosis and development of the disease. Thus, the objective of this study was to analyze the prevalence and social factors associated with rheumatic diseases and arthritis in the Brazilian population from a population-based health survey.

## Method

A cross-sectional, descriptive and analytical study was conducted based on secondary data which were produced from the National Health Survey (*PNS* Brazil) in 2013, developed by a conglomerate sample selected in three stages with simple random sampling: census sectors, fixed households and residents aged 18 or over. The *PNS* was approved by the National Research Ethics Commission (*CONEP*) under registration number 328,159 on June 26, 2013 and met all recommendations of Resolution 466/2012 of the National Health Council (*CNS*). All individuals were informed, clarified and voluntarily accepted to participate in the research.

Data collection took place from an analysis of the database in November 2019, and the final sample consisted of 60,202 individuals interviewed by trained researchers who answered the questions on the form in their home environment [14]. Additional information on the design of the *PNS* 2013 can be found in the National Health Research Report 2013, published by the Brazilian Institute of Geography and Statistics (*IBGE*) [15]. Although the PNS is a complex sample survey, with inclusion of sample variables and weight, these were not considered for association analysis due to the probabilities required for each level are not available in the database used by IBGE.

The dependent variable was assessed through the question (Q079): “Has any doctor already given you a diagnosis of arthritis or rheumatism?” with two answer options: yes or no.

The independent variables and their respective codes were sex (C006), age (C008), body mass index (BMI) which was calculated from the items weight (P00101) and height (P00401), stop performing usual activities (J002), what is your self-perception of health? (N001), physical activity (P034), and diagnosis of depression (Q092). The age variable was categorized into three ranges: adult individuals (18 to 34 years old), middle-aged (35 to 59 years old) and older adults (60 years old or more), while the BMI was classified as underweight (< 18.5), normal weight (between 18.5 and 24.9), overweight (between 25 and 29.9), and obesity (> 30).

A descriptive analysis of the data was initially performed and then association tests such as the Chi-squared test between the dependent variable and all the independent variables, estimating the prevalence ratios (PR) and their respective 95% confidence intervals (CI) in the unadjusted model. All independent variables which presented *p* ≤ 0.20 in the association test with the outcome variable were included in the multivariate regression model. The adjusted PR and 95% CI were obtained from the Poisson regression model. The analyzes were conducted using SPSS software version 22.0 and a significance level of 5% (α < 0.05).

## Results

This study covered the total sample of 60,202 individuals evaluated by the *PNS* in 2013. The number of individuals with rheumatic diseases or arthritis found in this study was 3853, corresponding to a prevalence of 6.4% for this population.

Table 1 shows the absolute and relative values for each of the independent variables analyzed in this study.

**Table 1:** Descriptive analysis of research participants

Variable	No. of people (%)
**Sex**	
Male	25,920 (43.1)
Female	34,282 (56.9)
**Age**	
18 to 34 years	21,746 (36.1)
35 to 59 years	27,279 (45.3)
60 or older	11,177 (18.6)
**BMI**	
Low weight	1372 (2.3)
Normal weight	23,379 (38.8)
Overweight	21,305 (35.4)
Obese	12,358 (20.5)
**Unable to perform habitual activities**	
No	54,935 (91.3)
Yes	5267 (8.7)
**Self-perception of health**	
Very good	7433 (12.3)
Good	31,708 (52.7)
Regular	17,197 (28.6)
Bad	3099 (5.1)
Very bad	765 (1.3)
**Practive physical activity**	
Yes	17,896 (29.7)
No	42,306 (70.3)
**Depression diagnosis**	
No	55,976 (93.0)
Yes	4235 (7.0)

*BMI* Body Mass Index

The associations between artrhitis and the independent variables analyzed are shown in Table 2. All variables included were associated with statistical significance (*p* ≤ 0.05).

**Table 2:** Relationship between the “Osteoarthritis” outcome and the independent variables

	Osteoarthritis		Non-adjusted			Adjusted
	Yes	No	***p***-value	PR (95%CI)	***p***-value	PR (95%CI)
	n (%)	n (%)				
**Sex**						
Male	25,007 (96.5)	913 (3.5)		1		1
Female	31,219 (91.1)	3063 (8.9)	< 0.001	2.54 (2.36–2.73)	< 0.001	2.09 (1.95–2.25)
**Age**						
18 to 34 years	21,398 (98.4)	3488 (1.6)		1		1
35 to 59 years	25,531 (93.6)	1748 (6.4)	< 0.001	4.00 (3.57–4.47)	< 0.001	2.88 (2.57–3.24)
60 or older	9297 (83.2)	1880 (16.8)	< 0.001	10.51 (9.40–11.76)	< 0.001	6.39 (5.68–7.19)
**BMI**						
Low weight	1422 (95.8)	62 (4.2)		1		
Normal weight	22,245 (95.1)	1134 (4.9)	0.244	1.17 (0.90–1.49)	0.006	1.42 (1.10–1.82)
Overweight	19,851 (93.2)	1454 (6.8)	< 0.001	1.64 (1.27–2.13)	< 0.001	1.61 (1.25–2.07)
Obese	11,366 (89.8)	1285 (10.2)	< 0.001	2.44 (1.89–3.17)	< 0.001	1.92 (1.50–2.47)
**Unable to perform habitual activities**						
No	51,884 (94.4)	3051 (5.6)		1		1
Yes	4342 (82.4)	925 (17.6)	< 0.001	3.16 (2.95–3.38)	< 0.001	1.61 (1.50–1.73)
**Self-perception of health**						
Very good	7279 (97.9)	154 (2.1)		1		1
Good	30,632 (96.6)	1076 (3.4)	< 0.001	1.64 (1.39–1.94)	< 0.001	1.41 (1.20–1.66)
Regular	15,259 (88.7)	1938 (11.3)	< 0.001	5.44 (4.63–6.39)	< 0.001	2.30 (2.54–3.52)
Bad	2475 (79.9)	624 (20.1)	< 0.001	9.72 (8.19–11.53)	< 0.001	3.96 (3.31–4.72)
Very bad	581 (75.9)	184 (24.1)	< 0.001	11.61 (9.50–14.19)	< 0.001	3.99 (3.24–4.90)
**Practice physical activity**						
Yes	16,964 (94.8)	932 (5.2)		1		1
No	39,262 (92.8)	3044 (7.2)	< 0.001	1.38 (1.29–1.48)	0.032	0.93 (0.86–0.99)
**Diagnosis of depression**						
No	57,782 (94.3)	3185 (5.7)		1		1
Yes	3444 (81.3)	791 (18.7)	< 0.001	3.28 (3.06–3.52)	< 0.001	1.77 (1.64–1.90)

*PR* Prevalence Ratio; *CI* Confidence Interval; *BMI* Body Mass Index

Among the analyzed independent variables, only not practicing physical activity was associated with a lower prevalence of rheumatic diseases or arthritis (PR = 0.93, 95% CI = 0.86–0.99). All other factors were associated with higher prevalence of arthritis.

The most intense associations were represented by the female, which is twice as prevalent (PR = 2.09, 95% CI = 1.95–2.25), while being in the middle-age range (35 to 59 years) increased the prevalence by almost three times (PR = 2.88, 95% CI = 2.57–3.24). Furthermore, being an older adult (60 years or older) increases the prevalence of the outcome by 6.4 times (PR = 6.39, 95% CI = 5.68–7.19), as well as having a poor perception of their own health (bad or very bad) increased artrhitis prevalence by approximately four times (PR = 3.96, 95% CI = 3.31–4.72; and PR = 3.99, 95% CI = 3.24–4.90), respectively. Presence of depression (PR = 1.77, 95% CI = 1.64–1.90) and obese BMI (PR = 1.92, (95% CI = 1.50–2.47) also showed a significant association with the presence of arthritis.

## Discussion

This study aimed to analyze the external factors associated with the prevalence of OA in the Brazilian population with a description of 60,202 individuals as assessed by the *PNS* in 2013. The results showed that most of the analyzed outcomes showed significant associations with the prevalence of OA, with greater representation in the female, in the middle-age range, performing less habitual activities and having poor self-perception of health.

The OA prevalence in this study was similar to that found in the National Household Sample Survey (*PNAD*) [16] in 2008, in which arthritis or rheumatism ranked third among chronic conditions, second only to arterial hypertension and back diseases [12]. This fact was also confirmed in a recent study [17].

Variables such as sex, age and weight are individual factors which are widely described in the literature [18–20]. Most studies confirm the prevalence of OA with a two-fold increase for women compared to men [21, 22] and at older ages [23], similar to what was found in this study. This fact may be linked to hormonal issues, especially when it occurs in middle age around 50 years, in which women go through the climacteric and menopause process and are more exposed to metabolic and joint disorders due to the decrease in the estrogen hormone [24, 25]. Another aspect which may be associated is the volume of articular cartilage, which is considerably lower in women [26]. Regarding age, it is known that the aging process generates a natural and progressive wear on the structures which compose the joints, leading to physical and functional consequences of individuals, as in osteoarthritis [23]. Such a progressive effect was confirmed by the gradual increase in the prevalence of OA with increasing age.

It was also observed in this study that OA showed a gradient of increasing prevalence of association with BMI. This finding is supported by a systematic review, which found a positive risk of high BMI for OA in all 36 studies which were part of the review [27]. Among the modifiable factors, it is known that overweight and obesity cause mechanical overload on the joints which support the body, especially the knees and hips, which are normally the most affected regions [28]. However, another study showed that the relationship of obesity in OA is beyond the biomechanical cause, showing that there is a direct influence of metabolic and inflammatory aspects that are present in these cases [29].

As already well documented in the literature, it was observed in this study that the presence of arthritis is strongly associated with females, advanced ages and excessive body weight. In addition to these, was observed that failure to perform habitual activities in the internal environment, such as domestic services and external activities such as going to work or shopping are associated with a higher prevalence of arthritis. This fact may be related to sedentary behavior and the consequent disuse of muscles and joints, which may accelerate the tissue degeneration process due to muscle weakness and immobility. On the other hand, after adjusting the multivariate model, lower levels of physical activity became a factor associated with a lower prevalence of arthritis. We believe that this phenomenon has occurred as a protective factor, in which individuals with arthritis avoid practicing physical activities for fear of generating more pain, with the consequence of these individuals’ low adherence to regular physical activity programs. This reduction, however, should be considered with caution, as in addition to being only 7%, it presented limit values for the confidence interval.

The prevalence of rheumatic diseases or arthritis was associated with a greater presence of comorbidities. According to a study [17], hypertension, diabetes, kidney problems and depression stood out as the most frequent. The latter was also associated with a higher prevalence of arthritis in the Brazilian population, suggesting that the multifactorial impact of the disease may influence mental health. Depression is a common comorbidity in rheumatic diseases and other chronic musculoskeletal diseases [30]. A population-based study, similar to this one, found a higher frequency of depression in individuals with self-reported arthritis [31]. In addition, higher depression scores are associated with lower levels of physical activity [32, 33]. This set of factors may be responsible for social isolation [34] and reduced mobility, generating a vicious cycle in conditions of pain, disability and low physical activity and affected mental health in individuals with rheumatic diseases.

The self-perception of the limitation degree shows a high level of agreement with the individual’s clinical condition [35]. In a study which classified the limitation degree as intense or very intense for habitual activities in chronic diseases, OA occupied the third position behind mental illness and stroke sequelae [11]. This fact reveals a general concern for society, as there is a general increase in the limitations of habitual activities in chronic diseases due to the reduced functional capacity and little knowledge about diagnostic and treatment methods. This generates a relevant social and economic impact [36].

The individual’s perception of feeling sick is not just a result of physical sensations, but of the social and psychological consequences of the disease [37]. The self-perceived health assessed in this study showed a strong concentration gradient for the categories bad and very bad, corroborating with a study [38] which evaluated the impact of osteoarthritis on the ability to work and activities of daily living, and showed a strong association with worse physical health; it is also related to reports of pain at higher levels.

Among the associated factors, age and sex target groups are at greater risk for developing this chronic condition and therefore need strategic health promotion actions and preventive measures such as screening for early diagnosis of this condition. However, modifiable factors should be considered such as overweight and poor health perception, as these factors can be changed or minimized through specific strategic and political actions, since they are not purely individual or biological factors, but involve a variety of health determinants and conditions. In addition, a sedentary lifestyle and immobility suggest isolation and consequent diagnosis of depression, making supportive measures relevant for greater social participation and physical activity.

In this study we can see that other factors are involved in OA, however the cross-sectional study design limits the analysis because it does not enable for an inference of reverse causality. Another limitation in this study was the diagnostic method adopted in this study following the *PNS* assessment instrument, in which it does not differentiate the type of arthritis or rheumatism pointed out by the interviewee. In addition, it would be pertinent to investigate other conditions and characteristics of the population with this disease, such as knowledge about the disease and measures used for treatment. Thus, it is suggested that prospective studies be developed to better manage cases and reduce the magnitude of social impacts on the lives of people with OA observed in this study.

## Conclusion

The factors that were associated with a higher prevalence of rheumatic diseases or arthritis in the Brazilian population were less practice of habitual activities, poor self-perception of their own health and diagnosis of depression, in addition to others already supported, such as female, aging and hight weight. Social determinants of health at this level can be modified based on public policies that provide incentives for regular physical activity, health education and, consequently, greater social participation.

## Data Availability

The data that support the findings of this study of the Health National Survey (PNS 2013) can be found in the National Health Research Report 2013, published by the Brazilian Institute of Geography and Statistics (IBGE).

## Abbreviations

OA: Osteoarthritis
PR: Prevalence Ratio
CI: Confidence Interval
NCDS: Chronic Non-Communicable Diseases
PNS: National Health Survey
CONEP: National Research Ethics Commission
CNS: National Health Council
STROBE: Strengthening the Reporting of Observational Studies in Epidemiology
IBGE: Brazilian Institute of Geography and Statistics
BMI: Body Mass Index
PNAD: National Household Sample Survey

## References

[1] Marmot M, Allen J, Bell R, Bloomer E, Goldblatt P (2012). WHO European review of social determinants of health and the health divide Consortium for the European Review of Social Determinants of Health and the Health Divide. Lancet.

[2] Vos T, Allen C, Arora M (2016). Global, regional, and national incidence, prevalence, and years lived with disability for 310 diseases and injuries, 1990–2015: a systematic analysis for the global burden of disease study 2015. Lancet.

[3] Conaghan PG (2008). Osteoarthritis: national clinical guideline for care and management in adults (NICE).

[4] Coimbra IB, Pastor EH, Greve JMD, et al. Consenso brasileiro para o tratamento da osteoartrite (artrose). Rev bras Reum. 2002:371–4.

[5] Zhang W, Nuki G, Moskowitz RW (2010). OARSI recommendations for the management of hip and knee osteoarthritis. Part III: changes in evidence following systematic cumulative update of research published through January 2009. Osteoarthr Cartil.

[6] Vasconcelos K, Dias J, Dias R (2006). Relação entre intensidade de dor e capacidade funcional em indivíduos obesos com osteoartrite de joelho. Rev Bras Fisioter.

[7] Brasil. Secretaria de Políticas de Previdência Social. Segundo boletim quadrimestral sobre benefícios por incapacidade [Internet]. Brasília, DF: Ministério da Previdência Social, 2013. [citado 2020 Mar 28]. Disponível em: http://www.previdencia.gov.br/wp-content/uploads/ 2015/01/2%C2%BA-boletim-quadrimestral.pdf.

[8] Dibonaventura MD, Gupta S, McDonald M (2011). Evaluating the health and economic impact of osteoarthritis pain in the workforce: results from the National Health and wellness survey. BMC Musculoskelet Disord.

[9] Wibelinger LM, Tombini DK (2010). Perfil epidemiológico dos pacientes atendidos no Serviço de Fisioterapia Reumatológica da Universidade de Passo Fundo. Rbceh.

[10] Kim Le T, Montejano LB, Cao Z, et al. Health care costs in US patients with and without a diagnosis of osteoarthritis. J Pain Res 2012; 5: 23–30. 10.2147/JPR.S27275 PMC3273404 22328832

[11] Theme Filha MM, de Souza Junior PRB, Damacena GN (2015). Prevalência de doenças crônicas não transmissíveis e associação com autoavaliação de saúde: Pesquisa nacional de saúde, 2013. Rev Bras Epidemiol.

[12] Malta DC, Bernal RTI, Lima MG (2017). Noncommunicable diseases and the use of health services: analysis of the National Health Survey in Brazil. Rev Saude Publica.

[13] de Azevedo Barros MB, Francisco PMSB, Zanchetta LM (2011). Tendências das desigualdades sociais e demográficas na prevalência de doenças crônicas no Brasil, PNAD: 2003-2008. Cienc e Saude Coletiva.

[14] Szwarcwald CL, Malta DC, Pereira CA (2014). Pesquisa nacional de saúde no Brasil: Concepção e metodologia de aplica ção. Cienc e Saude Coletiva.

[15] IBGE. Pesquisa Nacional de Saúde 2013 (2014). Percepção do estado de saúde, estilo de vida e doenças crônicas. Instituto Brasileiro de Geografia e Estatística (IBGE).

[16] IBGE (2010). Pesquisa Nacional por Amostra de Domicílios. Um panorama da saúde no Brasil: acesso e utilização dos serviços, condições de saúde e fatores de risco e proteção à saúde 2008.

[17] Malta DC, Stopa SR, Szwarcwald CL (2015). A vigilância e o monitoramento das principais doenças crônicas não transmissíveis no Brasil – pesquisa nacional de saúde, 2013. Rev Bras Epidemiol.

[18] Krasnokutsky S, Attur M, Palmer G (2008). Current concepts in the pathogenesis of osteoarthritis. Osteoarthr Cartil.

[19] Sakalauskiene G, Jauniškiene D (2010). Osteoarthritis: etiology, epidemiology, impact on the individual and society and the main principles of management. Medicina (B Aires).

[20] Zhang Y, Jordan JM (2010). Epidemiology of osteoarthritis. Clin Geriatr Med.

[21] Cunha-Miranda L, Faustino A, Alves C (2015). Avaliação da magnitude da desvantagem da osteoartrite na vida das pessoas: Estudo MOVES. Rev Bras Reumatol.

[22] Srikanth VK, Fryer JL, Zhai G (2005). A meta-analysis of sex differences prevalence, incidence and severity of osteoarthritis. Osteoarthr Cartil.

[23] Cho HJ, Chang CB, Kim KW (2011). Gender and prevalence of knee osteoarthritis types in elderly Koreans. J Arthroplast.

[24] Sowers MFR, McConnell D, Jannausch M (2006). Estradiol and its metabolites and their association with knee osteoarthritis. Arthritis Rheum.

[25] Hanna FS, Wluka AE, Bell RJ (2004). Osteoarthritis and the postmenopausal woman: epidemiological, magnetic resonance imaging, and radiological findings. Semin Arthritis Rheum.

[26] Cicuttini F, Forbes A, Morris K (1999). Gender differences in knee cartilage volume as measured by magnetic resonance imaging. Osteoarthr Cartil.

[27] Blagojevic M, Jinks C, Jeffery A (2010). Risk factors for onset of osteoarthritis of the knee in older adults: a systematic review and meta-analysis. Osteoarthr Cartil.

[28] Jiang L, Rong J, Wang Y (2011). The relationship between body mass index and hip osteoarthritis: a systematic review and meta-analysis. Joint Bone Spine.

[29] Sowers MR, Karvonen-Gutierrez CA (2010). The evolving role of obesity in knee osteoarthritis. Curr Opin Rheumatol.

[30] Lin EHB (2008). Depression and osteoarthritis. Am J Med.

[31] Hill CL, Gill T, Taylor AW, Daly A, Dal Grande E, Adams RJ (2007). Psychological factors and quality of life in arthritis: a population-based study. Clin Rheumatol.

[32] Schuch F, Vancampfort D, Firth J, Rosenbaum S, Ward P, Reichert T, Bagatini NC, Bgeginski R, Stubbs B (2017). Physical activity and sedentary behavior in people with major depressive disorder: a systematic review and meta-analysis. J Affect Disord.

[33] Schuch FB, Stubbs B (2019). The role of exercise in preventing and treating depression. Curr Sports Med Rep.

[34] Siviero P, Veronese N, Smith T, Stubbs B, Limongi F, Zambon S, Dennison EM, Edwards M, Cooper C, Timmermans EJ, van Schoor NM, van der Pas S, Schaap LA, Denkinger MD, Peter R, Herbolsheimer F, Otero A, Castell MV, Pedersen NL, Deeg DJH (2019). Maggi S: for the EPOSA research GroupAssociation between osteoarthritis and social isolation: data from the EPOSA study. J Am Geriatr Soc.

[35] Theme Filha MM, Szwarcwald CL, de Souza PRB (2008). Measurements of reported morbidity and interrelationships with health dimensions. Rev Saude Publica.

[36] Laires P, Gouveia M BJ. *O impacto econômico das doenças reumáticas.* Lisboa, http://ondor.med.up.pt/uploads/cap5.pdf (2007).

[37] Pavão ALB, Werneck GL, Campos MR (2013). Autoavaliação do estado de saúde e a associação com fatores sociodemográficos, hábitos de vida e morbidade na população: Um inquérito nacional. Cad Saude Publica.

[38] Cunha-Miranda LCT (2009). Rheumatic diseases and work: patient activity versus disease activity. Acta Reum Port.

